# Mortality Among Persons with Both Asthma and Chronic Obstructive Pulmonary Disease Aged ≥25 Years, by Industry and Occupation — United States, 1999–2016

**DOI:** 10.15585/mmwr.mm6922a3

**Published:** 2020-06-05

**Authors:** Katelynn E. Dodd, John Wood, Jacek M. Mazurek

**Affiliations:** 1 Respiratory Health Division, National Institute for Occupational Safety and Health, CDC.

Patients with asthma typically have chronic airway inflammation, variable airflow limitation, and intermittent respiratory symptoms; patients with chronic obstructive pulmonary disease (COPD) often have fixed airflow limitation and persistent respiratory symptoms. Some patients exhibit features suggesting that they have both conditions, which is termed asthma-COPD overlap. These patients have been reported to have worse health outcomes than do those with asthma or COPD alone (*1*). To describe mortality among persons aged ≥25 years with asthma-COPD overlap, CDC analyzed 1999–2016 National Vital Statistics multiple-cause-of-death mortality data* extracted from the National Occupational Mortality System (NOMS), which included industry and occupation^†^ information collected from 26 states^§^ for the years 1999, 2003, 2004, and 2007–2014. Age-adjusted death rates per one million persons^¶^ and proportionate mortality ratios (PMRs)** were calculated. During 1999–2016, 6,738 male decedents (age-adjusted rate per million = 4.30) and 12,028 female decedents (5.59) had both asthma and COPD assigned on their death certificate as the underlying or contributing cause of death. The annual age-adjusted death rate per million among decedents with asthma-COPD overlap declined from 6.70 in 1999 to 3.01 in 2016 (p<0.05) for men and from 7.71 in 1999 to 4.01 in 2016 (p<0.05) for women. Among adults aged 25–64 years, asthma-COPD overlap PMRs, by industry, were significantly elevated among nonpaid workers, nonworkers, and persons working at home for both men (1.72) and women (1.40) and among male food, beverage, and tobacco products workers (2.64). By occupation, asthma-COPD overlap PMRs were significantly elevated among both men (1.98) and women (1.79) who were unemployed, had never worked, or were disabled workers and among women bartenders (3.28) and homemakers (1.34). The association between asthma-COPD overlap mortality and nonworking status among adults aged 25–64 years suggests that asthma-COPD overlap might be associated with substantial morbidity. Increased risk for asthma-COPD overlap mortality among adults in certain industries and occupations suggests targets for public health interventions (e.g., elimination of or removal from exposures, engineering controls, and workplace smoke-free policies) to prevent asthma and COPD in and out of the workplace.

For this report, 1999–2016 National Vital Statistics System’s multiple-cause-of-death data extracted from NOMS were analyzed. Decedents with asthma-COPD overlap were identified using the *International Classification of Diseases, Tenth Revision* codes from death certificates for which both asthma and COPD^††^ were listed as the underlying or contributing cause of death. Death rates per million persons aged ≥25 years were assessed by sex and year and were age-adjusted using the 2000 U.S. Census standard population. Time trends in log-transformed age-adjusted mortality rates were assessed in Joinpoint software^§§^ by performing a sequence of permutation tests using Monte Carlo sampling and the Bonferroni correction for multiple testing. Information on industry and occupation, coded by the National Institute for Occupational Safety and Health using the U.S. Census 2000 Industry and Occupation Classification System, was available from 26 states for the years 1999, 2003, 2004, and 2007–2014.^¶¶^ PMRs, relative to the expected number of decedents with asthma-COPD overlap, and 95% confidence intervals (CIs) were generated by industry and occupation for men and women and adjusted for 5-year age groups and race. Joinpoint (version 4.7.0.0; National Cancer Institute) and SAS software (version 9.4; SAS Institute) were used to conduct all statistical analyses.

During 1999–2016, among U.S. decedents aged ≥25 years, a total of 4,689,828 had COPD and 164,731 had asthma assigned on their death certificate as the underlying or contributing cause of death. Among these decedents, 18,766 had both asthma and COPD assigned as the underlying or contributing cause of death (6,738 among men and 12,028 among women). The overall death rate among those with asthma-COPD overlap was 5.03 per million persons (4.30 among men and 5.59 among women). The annual age-adjusted death rate per million for men declined from 6.70 in 1999 to 3.01 in 2016 (annual percent change [APC] = –4.82%; p<0.05) and for women declined from 7.71 in 1999 to 4.01 in 2016 (APC = –3.63%; p<0.05) (Figure).

**FIGURE:**
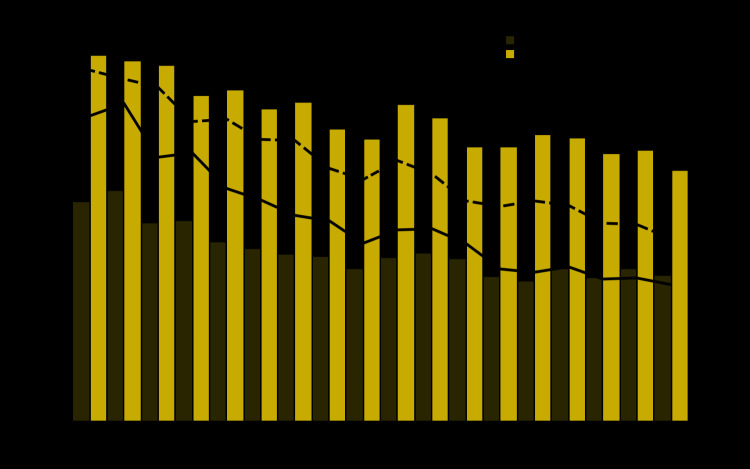
Number of asthma and chronic obstructive pulmonary disease (COPD) overlap deaths* and age-adjusted asthma-COPD overlap death rates^†^ among decedents aged ≥25 years, by sex — United States, 1999–2016 * Decedents with *International Classification of Diseases, Tenth Revision* codes for asthma: J45.0 (predominantly allergic asthma), J45.1 (nonallergic asthma), J45.8 (mixed asthma), J45.9 (asthma, unspecified), J46 (status asthmaticus); and COPD: J40 (bronchitis, not specified as acute or chronic), J41.0 (simple chronic bronchitis), J41.1 (mucopurulent chronic bronchitis), J41.8 (mixed simple and mucopurulent chronic bronchitis), J42 (unspecified chronic bronchitis), J43.0 (MacLeod's syndrome), J43.1 (panlobular emphysema), J43.2 (centrilobular emphysema), J43.8 (other emphysema), J43.9 (emphysema, unspecified), J44.0 (chronic obstructive pulmonary disease with acute lower respiratory infection), J44.1 (chronic obstructive pulmonary disease with acute exacerbation, unspecified), J44.8 (other specified chronic obstructive pulmonary disease), J44.9 (chronic obstructive pulmonary disease, unspecified) assigned as the underlying cause of death (i.e., the disease or injury which initiated the chain of morbid events leading directly to death, or the circumstances of the accident or violence which produced the fatal injury) or as a contributing cause of death. ^†^ Age-adjusted death rates per million persons were calculated by applying age-specific death rates to the 2000 U.S. Census standard population age distribution. https://wonder.cdc.gov/wonder/help/mcd.html#Age-AdjustedRates.

Among persons aged 25–64 years in 26 states during 1999, 2003, 2004, and 2007–2014, industry and occupation data were available for 784 (99.1%) of 791 decedents with asthma-COPD overlap (314 [99.4%] of 316 men and 470 [98.9%] of 475 women). By industry, asthma-COPD overlap PMRs were significantly elevated among nonpaid workers, nonworkers, and persons working at home for both men (1.72) and women (1.40) and among male food, beverage, and tobacco products workers (2.64) (Table 1). By occupation, asthma-COPD overlap PMRs were significantly elevated among men (1.98) and women (1.79) who were unemployed, never worked, or were disabled workers and among women bartenders (3.28) and homemakers (1.34) (Table 2).

**TABLE 1:** Industries with five or more asthma-COPD overlap deaths* among decedents aged ≥25 years, by sex and age group — 26 states,^†^ 1999, 2003, 2004, and 2007–2014

Industry	Decedents aged 25–64 yrs		Decedents aged ≥65 yrs	
	Deaths	PMR^§^ (95% CI)	Deaths	PMR^§^ (95% CI)
**Male**				
Computer and electronic products^¶^	N/A	N/A	10	2.58 (1.24–4.74)**
Lumber, wood products, and furniture^¶^	N/A	N/A	12	2.53 (1.30–4.41)**
Sawmills and wood preservation^††^	N/A	N/A	5	3.57 (1.16–8.34)**
Agriculture, forestry, fishing and hunting^¶^	N/A	N/A	66	1.82 (1.42–2.33)**
Logging^††^	N/A	N/A	11	4.82 (2.41–8.62)**
Animal production^††^	N/A	N/A	16	1.63 (0.93–2.65)
Crop production^††^	N/A	N/A	35	1.59 (1.10–2.21)**
Broadcasting and telecommunications^¶^	N/A	N/A	11	1.40 (0.70–2.50)
Wired telecommunications carriers^††^	N/A	N/A	8	1.51 (0.65–2.97)
Personal and laundry services^¶^	N/A	N/A	7	1.37 (0.55–2.82)
Nonmetallic mineral products^¶^	N/A	N/A	5	1.30 (0.42–3.04)
Wholesale trade^¶^	N/A	N/A	16	1.24 (0.71–2.01)
Groceries and related product wholesalers^††^	N/A	N/A	5	1.99 (0.64–4.65)
Paper and printing^¶^	N/A	N/A	9	1.20 (0.55–2.29)
Printing and related support activities^††^	N/A	N/A	6	1.56 (0.57–3.39)
Publishing, and motion picture and sound recording industries^¶^	N/A	N/A	5	1.16 (0.37–2.70)
Primary metal industries^¶^	N/A	N/A	14	1.09 (0.60–1.83)
Iron and steel mills and steel product manufacturing^††^	N/A	N/A	10	1.07 (0.52–1.97)
Utilities^¶^	N/A	N/A	11	1.03 (0.52–1.84)
Food, beverage, and tobacco products^¶^	10	2.64 (1.27–4.86)**	12	1.11 (0.57–1.93)
Beverage manufacturing^††^	N/A	N/A	5	3.15 (1.02–7.37)**
Arts, entertainment and recreation^¶^	N/A	N/A	6	0.98 (0.36–2.12)
Retired, unemployed, or nonpaid worker^¶^	38	1.71 (1.21–2.35)**	11	0.94 (0.47–1.68)
Nonpaid worker or nonworker or own home/at home^††^	38	1.72 (1.22–2.36)**	11	0.98 (0.49–1.75)
Unknown or not reported^¶^	24	1.50 (0.96–2.24)	18	1.18 (0.70–1.86)
Mining^¶^	N/A	N/A	11	0.90 (0.45–1.61)
Oil and gas extraction^††^	N/A	N/A	6	1.91 (0.70–4.16)
Finance and Insurance^¶^	N/A	N/A	13	0.86 (0.46–1.46)
Banking and related activities^††^	N/A	N/A	5	1.28 (0.42–3.00)
Insurance carriers and related activities^††^	N/A	N/A	6	0.77 (0.28–1.67)
Home furnishings, appliances, building materials, hardware, lawn and garden^¶^	5	1.49 (0.48–3.48)	5	0.60 (0.19–1.40)
Motor vehicle and parts dealers^¶^	6	1.44 (0.53–3.14)	5	0.58 (0.19–1.36)
Automobile dealers^††^	5	1.94 (0.63–4.53)	N/A	N/A
Repair and maintenance^¶^	16	1.35 (0.77–2.19)	15	0.94 (0.53–1.55)
Automotive repair and maintenance^††^	12	1.36 (0.70–2.37)	9	0.80 (0.37–1.51)
Military^¶^	5	1.29 (0.42–3.02)	10	0.68 (0.32–1.24)
Other retail trade^¶^	12	1.24 (0.64–2.17)	21	1.04 (0.64–1.59)
Gasoline stations^††^	N/A	N/A	5	2.50 (0.81–5.83)
Not specified retail trade^††^	6	1.32 (0.48–2.87)	5	0.67 (0.22–1.56)
Chemical^¶^	N/A	N/A	7	0.79 (0.32–1.63)
Industrial and miscellaneous chemicals^††^	N/A	N/A	5	0.85 (0.27–1.97)
Food and beverage stores^¶^	N/A	N/A	6	0.75 (0.28–1.64)
Construction^¶^	58	1.16 (0.89–1.51)	73	1.08 (0.85–1.37)
Public administration^¶^	15	1.07 (0.60–1.76)	41	1.00 (0.72–1.36)
Other general government and support^††^	5	0.95 (0.31–2.23)	16	0.97 (0.56–1.58)
Justice, public order, and safety activities^††^	5	0.94 (0.30–2.19)	9	0.73 (0.33–1.38)
Health care^¶^	10	1.05 (0.51–1.94)	11	0.74 (0.37–1.32)
Hospitals^††^	5	1.17 (0.38–2.72)	7	1.19 (0.48–2.46)
Miscellaneous manufacturing^¶^	11	0.90 (0.45–1.61)	41	1.39 (1.00–1.89)**
Not specified manufacturing industries^††^	11	0.99 (0.50–1.77)	38	1.38 (0.98–1.90)
Educational services^¶^	7	0.83 (0.33–1.71)	22	0.85 (0.53–1.28)
Elementary and secondary schools^††^	N/A	N/A	17	0.91 (0.53–1.45)
Colleges and universities, including junior colleges^††^	N/A	N/A	5	0.78 (0.25–1.81)
Transportation and warehousing^¶^	19	0.76 (0.46–1.18)	53	1.01 (0.77–1.34)
Truck transportation^††^	15	1.17 (0.65–1.92)	22	1.14 (0.71–1.72)
Water transportation^††^	N/A	N/A	5	2.73 (0.88–6.38)
Postal service^††^	N/A	N/A	8	0.92 (0.40–1.81)
Professional, scientific, technical and management services^¶^	6	0.62 (0.23–1.35)	15	0.72 (0.40–1.18)
Machinery^¶^	N/A	N/A	6	0.72 (0.26–1.56)
Accommodation and food services^¶^	8	0.62 (0.27–1.22)	7	0.56 (0.23–1.16)
Restaurants and other food services^††^	7	0.69 (0.28–1.42)	5	0.56 (0.18–1.32)
Administrative and support, and waste management services^¶^	7	0.58 (0.23–1.20)	10	0.87 (0.42–1.60)
Transportation equipment^¶^	6	0.54 (0.20–1.18)	17	0.48 (0.28–0.76)
Motor vehicles and motor vehicle equipment manufacturing^††^	5	0.62 (0.20–1.45)	14	0.57 (0.31–0.95)
All other industries^¶^	51	N/A	22	N/A
**Female**				
Retired, unemployed, or nonpaid worker^¶^	192	1.40 (1.21–1.62)**	532	1.06 (0.97–1.15)
Nonpaid worker or nonworker or own home/at home^††^	192	1.40 (1.22–1.62)**	529	1.05 (0.96–1.15)
Private households^¶^	8	1.34 (0.58–2.63)	24	1.69 (1.08–2.51)**
Home furnishings, appliances, building materials, hardware, lawn and garden^¶^	N/A	N/A	10	1.69 (0.81–3.11)
Furniture and home furnishings stores^††^	N/A	N/A	6	2.99 (1.10–6.52)**
Machinery^¶^	N/A	N/A	5	1.53 (0.49–3.57)
Food, beverage, and tobacco products^¶^	N/A	N/A	14	1.25 (0.68–2.09)
Not specified food industries^††^	N/A	N/A	5	3.72 (1.20–8.68)**
Paper and printing^¶^	N/A	N/A	7	1.21 (0.49–2.50)
Utilities^¶^	N/A	N/A	5	1.14 (0.37–2.66)
Agriculture, forestry, fishing and hunting^¶^	N/A	N/A	11	1.13 (0.57–2.02)
Crop production^††^	N/A	N/A	7	1.14 (0.46–2.36)
Textile mill, apparel and other finished textile products^¶^	N/A	N/A	26	1.13 (0.74–1.65)
Cut and sew apparel manufacturing^††^	N/A	N/A	16	1.14 (0.65–1.84)
Fabric mills, except knitting^††^	N/A	N/A	6	0.91 (0.33–1.99)
Electrical equipment, appliances, and components^¶^	N/A	N/A	5	1.08 (0.35–2.51)
Publishing, and motion picture and sound recording industries^¶^	N/A	N/A	6	1.01 (0.37–2.19)
Administrative and support, and waste management services^¶^	13	1.25 (0.66–2.13)	12	0.72 (0.37–1.26)
Business support services^††^	5	1.88 (0.61–4.39)	5	0.86 (0.28–2.01)
Arts, entertainment and recreation^¶^	6	1.10 (0.40–2.40)	6	0.65 (0.24–1.41)
Independent artists, performing arts, spectator sports, and related industries^††^	5	2.24 (0.73–5.24)	N/A	N/A
Unknown or not reported^¶^	16	1.09 (0.63–1.78)	19	0.90 (0.54–1.41)
Broadcasting and telecommunications^¶^	5	1.09 (0.35–2.54)	13	0.92 (0.49–1.57)
Wired telecommunications carriers^††^	N/A	N/A	10	0.92 (0.44–1.68)
Transportation equipment^¶^	N/A	N/A	15	0.87 (0.49–1.44)
Motor vehicles and motor vehicle equipment manufacturing^††^	N/A	N/A	10	0.86 (0.42–1.59)
Chemical^¶^	N/A	N/A	5	0.82 (0.26–1.91)
Food and beverage stores^¶^	6	1.00 (0.37–2.18)	19	1.34 (0.81–2.10)
Grocery stores^††^	6	1.09 (0.40–2.36)	15	1.19 (0.67–1.97)
Other retail trade^¶^	22	0.97 (0.61–1.47)	50	0.77 (0.57–1.01)
Clothing and accessories, except shoe, stores^††^	N/A	N/A	5	0.87 (0.28–2.04)
Department stores^††^	N/A	N/A	8	0.85 (0.37–1.68)
Not specified retail trade^††^	12	0.99 (0.51–1.73)	19	0.60 (0.36–0.94)
Health care^¶^	62	0.97 (0.75–1.26)	114	0.99 (0.82–1.20)
Outpatient care centers^††^	11	0.91 (0.45–1.63)	22	1.06 (0.66–1.60)
Other health care services^††^	9	1.40 (0.64–2.66)	10	1.04 (0.50–1.92)
Hospitals^††^	26	0.96 (0.63–1.41)	59	1.00 (0.77–1.30)
Nursing care facilities^††^	9	1.15 (0.53–2.19)	10	0.92 (0.44–1.69)
Miscellaneous manufacturing^¶^	10	0.95 (0.46–1.75)	41	1.25 (0.90–1.70)
Not specified manufacturing industries^††^	9	0.98 (0.45–1.86)	37	1.26 (0.89–1.73)
Real estate and rental leasing^¶^	5	0.93 (0.30–2.18)	13	0.97 (0.52–1.67)
Real estate^††^	5	0.98 (0.32–2.28)	13	0.99 (0.53–1.70)
Transportation and warehousing^¶^	9	0.87 (0.40–1.64)	14	0.81 (0.44–1.36)
Truck transportation^††^	N/A	N/A	6	1.91 (0.70–4.16)
Postal service^††^	N/A	N/A	5	1.06 (0.34–2.47)
Social assistance^¶^	7	0.83 (0.33–1.70)	8	0.78 (0.33–1.53)
Accommodation and food services^¶^	22	0.82 (0.51–1.24)	58	1.17 (0.90–1.52)
Restaurants and other food services^††^	15	0.72 (0.40–1.18)	51	1.27 (0.96–1.69)
Traveler accommodation^††^	N/A	N/A	5	0.69 (0.22–1.61)
Personal and laundry services^¶^	7	0.81 (0.33–1.67)	23	1.12 (0.71–1.68)
Beauty salons^††^	7	1.24 (0.50–2.55)	14	1.04 (0.57–1.74)
Drycleaning and laundry services^††^	N/A	N/A	7	1.47 (0.59–3.03)
Professional, scientific, technical and management services^¶^	12	0.80 (0.41–1.39)	24	0.88 (0.56–1.30)
Legal services^††^	N/A	N/A	8	1.19 (0.51–2.33)
Accounting, tax preparation, bookkeeping and payroll services^††^	5	1.31 (0.42–3.05)	7	0.75 (0.30–1.54)
Public administration^¶^	13	0.74 (0.39–1.27)	49	1.09 (0.81–1.45)
National security and international affairs^††^	N/A	N/A	5	1.44 (0.47–3.36)
Administration of human resource programs^††^	N/A	N/A	5	1.38 (0.45–3.22)
Justice, public order, and safety activities^††^	N/A	N/A	6	0.87 (0.32–1.89)
Other general government and support^††^	8	1.04 (0.45–2.05)	26	1.09 (0.71–1.60)
Finance and insurance^¶^	9	0.62 (0.29–1.18)	28	0.79 (0.53–1.15)
Insurance carriers and related activities^††^	5	0.85 (0.28–2.00)	8	0.60 (0.26–1.19)
Banking and related activities^††^	N/A	N/A	14	0.87 (0.47–1.46)
Educational services^¶^	19	0.61 (0.37–0.96)	86	0.87 (0.70–1.08)
Elementary and secondary schools^††^	18	0.68 (0.40–1.07)	69	0.80 (0.62–1.01)
Colleges and universities, including junior colleges^††^	N/A	N/A	16	1.71 (0.98–2.78)
Wholesale trade^¶^	N/A	N/A	5	0.75 (0.24–1.75)
All other industries^¶^	27	N/A	37	N/A

**Abbreviations:** CI = confidence interval; N/A = not applicable; PMR = proportionate mortality ratio.

* Decedents with *International Classification of Diseases, Tenth Revision* codes for asthma: J45.0 (predominantly allergic asthma), J45.1 (nonallergic asthma), J45.8 (mixed asthma), J45.9 (asthma, unspecified), J46 (status asthmaticus); and COPD: J40 (bronchitis, not specified as acute or chronic), J41.0 (simple chronic bronchitis), J41.1 (mucopurulent chronic bronchitis), J41.8 (mixed simple and mucopurulent chronic bronchitis), J42 (unspecified chronic bronchitis), J43.0 (MacLeod's syndrome), J43.1 (panlobular emphysema), J43.2 (centrilobular emphysema), J43.8 (other emphysema), J43.9 (emphysema, unspecified), J44.0 (chronic obstructive pulmonary disease with acute lower respiratory infection), J44.1 (chronic obstructive pulmonary disease with acute exacerbation, unspecified), J44.8 (other specified chronic obstructive pulmonary disease), J44.9 (chronic obstructive pulmonary disease, unspecified) assigned as the underlying cause of death (i.e., the disease or injury which initiated the chain of morbid events leading directly to death, or the circumstances of the accident or violence which produced the fatal injury) or as a contributing cause of death.

^†^ Colorado, Florida, Georgia, Hawaii, Idaho, Indiana, Kansas, Kentucky, Louisiana, Michigan, Nebraska, Nevada, New Hampshire, New Jersey, New Mexico, North Carolina, North Dakota, Ohio, Rhode Island, South Carolina, Texas, Utah, Vermont, Washington, West Virginia, and Wisconsin.

^§^ PMR was defined as the observed number of deaths from asthma-COPD overlap in a specified industry or occupation, divided by the expected number of deaths from asthma-COPD overlap. The expected number of deaths was the total number of deaths in industry or occupation of interest multiplied by a proportion defined as the number of asthma-COPD overlap deaths in all industries or occupations, divided by the total number of deaths in all industries or occupations. The asthma-COPD overlap PMRs were internally adjusted by 5-year age groups, sex, and race. CIs were calculated assuming Poisson distribution of the data. A PMR >1.0 indicates that there were more deaths associated with the condition in a specified occupation or industry than expected; a PMR <1.0 indicates that there were fewer deaths associated with the condition in a specified occupation or industry than expected.

^¶^ U.S. Census 2000 Industry Classification System two-digit industries with five or more deaths.

** Statistically significantly elevated PMR.

^††^ U.S. Census 2000 Industry Classification System three-digit industry groups with five or more deaths.

**TABLE 2:** Occupations with five or more asthma-COPD overlap deaths* among decedents aged ≥25 years, by sex and age group — 26 states,^†^ 1999, 2003, 2004, 2007–2014

Occupation	Decedents aged 25–64 yrs		Decedents aged ≥65 yrs	
	Deaths	PMR^§^ (95% CI)	Deaths	PMR^§^ (95% CI)
**Male**				
Fishing, hunting, and forestry occupations^¶^	N/A	N/A	10	3.78 (1.82–6.95)**
Logging workers^††^	N/A	N/A	10	5.64 (2.71–10.37)**
Farmers and farm managers^¶^	N/A	N/A	43	1.62 (1.17–2.18)**
Farmers and ranchers^††^	N/A	N/A	43	1.67 (1.21–2.25)**
Food processing workers^¶^	N/A	N/A	6	1.59 (0.58–3.47)
Textile, apparel, and furnishings workers^¶^	N/A	N/A	6	1.56 (0.57–3.39)
Retired, students, volunteers, homemakers and unemployed^¶^	40	1.77 (1.26–2.41)**	11	0.89 (0.44–1.59)
Unemployed, never worked, disabled^††^	36	1.98 (1.39–2.75)**	8	1.38 (0.59–2.71)
Vehicle and mobile equipment mechanics, installers, and repairers^¶^	15	1.41 (0.79–2.33)	17	1.06 (0.62–1.69)
Automotive service technicians and mechanics^††^	7	1.03 (0.41–2.13)	6	0.71 (0.26–1.54)
Unknown or not reported^¶^	18	1.30 (0.77–2.05)	15	0.97 (0.54–1.60)
Metal workers and plastic workers^¶^	13	1.27 (0.67–2.17)	25	0.93 (0.60–1.37)
Welding, soldering, and brazing workers^††^	5	1.31 (0.42–3.06)	N/A	N/A
Tool and die makers^††^	N/A	N/A	5	1.32 (0.43–3.09)
Metalworkers and plastic workers, all other^††^	N/A	N/A	6	2.45 (0.90–5.34)
Machinists^††^	N/A	N/A	10	0.99 (0.48–1.83)
Laborers and material movers, hand^¶^	21	1.26 (0.78–1.92)	35	1.54 (1.07–2.14)**
Laborers and freight, stock, and material movers, hand^††^	21	1.34 (0.82–2.04)	32	1.47 (1.01–2.08)**
Agricultural workers, including supervisors^¶^	N/A	N/A	7	1.53 (0.62–3.16)
Miscellaneous agricultural workers^††^	N/A	N/A	6	1.58 (0.58–3.43)
Rail and water transportation workers^¶^	N/A	N/A	7	1.48 (0.59–3.04)
Motor vehicle operators^¶^	23	1.19 (0.75–1.78)	38	1.22 (0.86–1.67)
Bus drivers^††^	N/A	N/A	5	1.77 (0.57–4.12)
Driver-sales workers and truck drivers^††^	21	1.24 (0.76–1.89)	32	1.19 (0.81–1.68)
Other material moving workers, except laborers^¶^	N/A	N/A	7	1.21 (0.49–2.50)
Other production occupations, including supervisors^¶^	13	1.17 (0.62–2.00)	23	0.73 (0.46–1.10)
First-line supervisors or managers of production and operating workers^††^	N/A	N/A	9	0.83 (0.38–1.58)
Production workers, all other^††^	6	1.27 (0.47–2.77)	7	0.70 (0.28–1.44)
Construction trades workers^¶^	45	1.11 (0.81–1.48)	66	1.20 (0.94–1.54)
Carpenters^††^	10	1.23 (0.59–2.27)	20	1.68 (1.02–2.59)**
Operating engineers and other construction equipment operators^††^	N/A	N/A	8	1.59 (0.69–3.13)
Construction laborers^††^	17	1.29 (0.75–2.07)	16	1.49 (0.85–2.41)
Electricians^††^	7	1.93 (0.78–3.98)	N/A	N/A
Financial specialists^¶^	N/A	N/A	12	1.18 (0.61–2.06)
Accountants and auditors^††^	N/A	N/A	9	1.23 (0.57–2.34)
Business operations specialists^¶^	N/A	N/A	8	1.14 (0.49–2.24)
Drafters, engineering, and mapping technicians^¶^	N/A	N/A	5	1.07 (0.35–2.51)
Other protective service workers, including supervisors^¶^	N/A	N/A	5	1.03 (0.33–2.40)
Assemblers and fabricators^¶^	N/A	N/A	8	1.03 (0.44–2.02)
Miscellaneous assemblers and fabricators^††^	N/A	N/A	5	1.03 (0.33–2.40)
Law enforcement workers, including supervisors^¶^	N/A	N/A	8	1.02 (0.44–2.01)
Police and sheriff's patrol officers^††^	N/A	N/A	5	1.00 (0.33–2.35)
Extraction workers^¶^	N/A	N/A	6	1.01 (0.37–2.19)
Health diagnosing and treating practitioners and technical occupations^¶^	N/A	N/A	5	1.00 (0.32–2.34)
Engineers^¶^	N/A	N/A	22	0.95 (0.59–1.44)
Civil engineers^††^	N/A	N/A	6	1.43 (0.52–3.11)
Office and administrative support occupations^¶^	12	1.04 (0.54–1.82)	23	0.87 (0.55–1.30)
Building and grounds cleaning and maintenance occupations^¶^	15	1.03 (0.58–1.70)	18	0.92 (0.55–1.46)
Janitors and building cleaners^††^	12	1.38 (0.71–2.40)	12	0.85 (0.44–1.48)
Education, training, and library occupations^¶^	N/A	N/A	13	0.86 (0.46–1.48)
Postsecondary teachers^††^	N/A	N/A	5	1.20 (0.39–2.81)
Elementary and middle school teachers^††^	N/A	N/A	8	1.01 (0.43–1.98)
Supervisors, construction and extraction workers^¶^	N/A	N/A	6	0.78 (0.29–1.70)
First-line supervisors or managers of construction trades and extraction workers^††^	N/A	N/A	6	0.78 (0.29–1.70)
Electrical equipment mechanics and other installation, maintenance, and repair workers^¶^	9	1.01 (0.46–1.91)	16	0.75 (0.43–1.22)
First-line supervisors or managers of mechanics, installers, and repairers^††^	N/A	N/A	7	1.63 (0.65–3.35)
Food preparation and serving related occupations^¶^	7	0.77 (0.31–1.60)	N/A	N/A
Sales and related occupations^¶^	14	0.70 (0.38–1.17)	48	0.88 (0.65–1.17)
First-line supervisors or managers of nonretail sales workers^††^	N/A	N/A	6	1.20 (0.44–2.61)
Retail salespersons^††^	5	0.85 (0.27–1.98)	9	0.77 (0.35–1.46)
Sales representatives, wholesale and manufacturing^††^	N/A	N/A	5	0.77 (0.25–1.80)
First-line supervisors or managers of retail sales workers^††^	N/A	N/A	13	0.72 (0.38–1.23)
Management occupations, except agricultural^¶^	11	0.53 (0.26–0.94)	54	0.91 (0.69–1.19)
Managers, all other^††^	6	1.19 (0.44–2.60)	17	1.10 (0.64–1.77)
Industrial production managers^††^	N/A	N/A	10	2.23 (1.07–4.10)**
Transportation, storage, and distribution managers^††^	N/A	N/A	5	1.87 (0.60–4.36)
Chief executives^††^	N/A	N/A	8	1.55 (0.67–3.05)
Military occupations^¶^	N/A	N/A	9	0.66 (0.30–1.26)
Military, rank not specified^††^	N/A	N/A	6	1.06 (0.39–2.30)
All other occupations^¶^	58	N/A	42	N/A
**Female**				
Agricultural workers, including supervisors^¶^	N/A	N/A	5	2.00 (0.65–4.68)
Media and communications workers^¶^	N/A	N/A	5	1.41 (0.46–3.29)
Other protective service workers, including supervisors^¶^	5	2.34 (0.76–5.47)	N/A	N/A
Other production occupations, including supervisors^¶^	N/A	N/A	39	1.29 (0.92–1.77)
Inspectors, testers, sorters, samplers, and weighers^††^	N/A	N/A	8	1.19 (0.51–2.35)
Production workers, all other^††^	N/A	N/A	22	1.66 (1.04–2.52)**
Textile, apparel, and furnishings workers^¶^	N/A	N/A	34	1.20 (0.83–1.68)
Sewing machine operators^††^	N/A	N/A	21	1.32 (0.82–2.02)
Tailors, dressmakers, and sewers^††^	N/A	N/A	5	1.15 (0.37–2.68)
Retired, students, volunteers, homemakers and unemployed^¶^	193	1.41 (1.22–1.63)**	535	1.06 (0.97–1.15)
Unemployed, never worked, disabled^††^	41	1.79 (1.29–2.42)**	11	1.27 (0.64–2.27)
Homemakers^††^	151	1.34 (1.14–1.58)**	521	1.05 (0.97–1.15)
Farmers and farm managers^¶^	N/A	N/A	5	1.02 (0.33–2.38)
Farmers and ranchers^††^	N/A	N/A	5	1.07 (0.35–2.49)
Financial specialists^¶^	N/A	N/A	14	1.00 (0.54–1.67)
Accountants and auditors^††^	N/A	N/A	8	0.87 (0.38–1.72)
Health technologists and technicians^¶^	11	1.33 (0.66–2.38)	9	0.66 (0.30–1.26)
Licensed practical and licensed vocational nurses^††^	8	2.24 (0.96–4.40)	N/A	N/A
Healthcare support occupations^¶^	27	1.27 (0.83–1.84)	38	1.38 (0.98–1.89)
Nursing, psychiatric, and home health aides^††^	25	1.52 (0.98–2.25)	31	1.42 (0.97–2.02)
Motor vehicle operators^¶^	6	1.25 (0.46–2.73)	N/A	N/A
Sales and related occupations^¶^	31	1.00 (0.68–1.42)	66	0.83 (0.65–1.06)
Cashiers^††^	9	1.61 (0.74–3.06)	N/A	N/A
Retail salespersons^††^	11	1.12 (0.56–2.00)	35	1.08 (0.75–1.50)
First-line supervisors or managers of retail sales workers^††^	N/A	N/A	17	0.85 (0.49–1.35)
Building and grounds cleaning and maintenance occupations^¶^	14	0.96 (0.53–1.62)	34	1.13 (0.79–1.58)
Janitors and building cleaners^††^	6	1.41 (0.52–3.07)	9	1.16 (0.53–2.20)
Maids and housekeeping cleaners^††^	8	0.90 (0.39–1.76)	25	1.23 (0.79–1.81)
Personal care and service occupations^¶^	16	0.94 (0.54–1.53)	24	0.87 (0.56–1.29)
Personal and home care aides^††^	5	1.27 (0.41–2.97)	5	1.07 (0.34–2.49)
Hairdressers, hairstylists, and cosmetologists^††^	6	1.16 (0.42–2.52)	13	1.06 (0.56–1.81)
Community and social services occupations^¶^	6	0.92 (0.34–2.01)	N/A	N/A
Laborers and material movers, hand^¶^	8	0.92 (0.40–1.82)	25	1.31 (0.85–1.94)
Laborers and freight, stock, and material movers, hand^††^	8	1.01 (0.43–1.98)	25	1.51 (0.97–2.22)
Unknown or not reported^¶^	13	0.92 (0.49–1.57)	23	1.15 (0.73–1.73)
Food preparation and serving related occupations^¶^	18	0.83 (0.49–1.31)	57	1.22 (0.93–1.60)
Cooks^††^	6	0.91 (0.33–1.98)	23	1.26 (0.80–1.89)
Bartenders^††^	6	3.28 (1.20–7.15)**	N/A	N/A
Waiters and waitresses^††^	N/A	N/A	23	1.70 (1.07–2.55)**
Education, training, and library occupations^¶^	17	0.82 (0.48–1.31)	58	0.87 (0.67–1.14)
Elementary and middle school teachers^††^	10	0.82 (0.39–1.50)	40	0.86 (0.62–1.18)
Health diagnosing and treating practitioners and technical occupations^¶^	12	0.72 (0.37–1.25)	43	1.09 (0.79–1.46)
Registered nurses^††^	11	0.75 (0.38–1.34)	41	1.11 (0.80–1.50)
Office and administrative support occupations^¶^	36	0.64 (0.45–0.88)	152	0.86 (0.73–1.01)
First-line supervisors or managers of office and administrative support workers^††^	5	0.96 (0.31–2.24)	10	0.85 (0.41–1.57)
Secretaries and administrative assistants^††^	10	0.68 (0.33–1.26)	54	0.82 (0.62–1.08)
Office clerks, general^††^	7	1.04 (0.42–2.15)	18	0.85 (0.50–1.35)
Receptionists and information clerks^††^	N/A	N/A	6	1.24 (0.45–2.69)
Telephone operators^††^	N/A	N/A	7	1.18 (0.47–2.43)
Bookkeeping, accounting, and auditing clerks^††^	N/A	N/A	24	0.89 (0.57–1.32)
Office and administrative support workers, all other^††^	N/A	N/A	6	1.80 (0.66–3.92)
Management occupations, except agricultural^¶^	11	0.45 (0.23–0.81)	54	1.06 (0.80–1.39)
Property, real estate, and community association managers^††^	N/A	N/A	6	2.02 (0.74–4.40)
Food service managers^††^	N/A	N/A	10	1.14 (0.55–2.10)
Financial managers^††^	N/A	N/A	5	0.95 (0.31–2.22)
Managers, all other^††^	N/A	N/A	9	0.74 (0.34–1.41)
Business operations specialists^¶^	N/A	N/A	8	0.74 (0.32–1.45)
Assemblers and fabricators^¶^	N/A	N/A	5	0.36 (0.12–0.84)
All other occupations^¶^	46	N/A	51	N/A

**Abbreviations:** CI = confidence interval; N/A = not applicable; PMR = proportionate mortality ratio.

* Decedents with *International Classification of Diseases, Tenth Revision* codes for asthma: J45.0 (predominantly allergic asthma), J45.1 (nonallergic asthma), J45.8 (mixed asthma), J45.9 (asthma, unspecified), J46 (status asthmaticus); and COPD: J40 (bronchitis, not specified as acute or chronic), J41.0 (simple chronic bronchitis), J41.1 (mucopurulent chronic bronchitis), J41.8 (mixed simple and mucopurulent chronic bronchitis), J42 (unspecified chronic bronchitis), J43.0 (MacLeod's syndrome), J43.1 (panlobular emphysema), J43.2 (centrilobular emphysema), J43.8 (other emphysema), J43.9 (emphysema, unspecified), J44.0 (chronic obstructive pulmonary disease with acute lower respiratory infection), J44.1 (chronic obstructive pulmonary disease with acute exacerbation, unspecified), J44.8 (other specified chronic obstructive pulmonary disease), J44.9 (chronic obstructive pulmonary disease, unspecified) assigned as the underlying cause of death (i.e., the disease or injury which initiated the chain of morbid events leading directly to death, or the circumstances of the accident or violence which produced the fatal injury) or as a contributing cause of death.

^†^ Colorado, Florida, Georgia, Hawaii, Idaho, Indiana, Kansas, Kentucky, Louisiana, Michigan, Nebraska, Nevada, New Hampshire, New Jersey, New Mexico, North Carolina, North Dakota, Ohio, Rhode Island, South Carolina, Texas, Utah, Vermont, Washington, West Virginia, and Wisconsin.

^§^ PMR was defined as the observed number of deaths from asthma-COPD overlap in a specified industry or occupation, divided by the expected number of deaths from asthma-COPD overlap. The expected number of deaths was the total number of deaths in industry or occupation of interest multiplied by a proportion defined as the number of asthma-COPD overlap deaths in all industries or occupations, divided by the total number of deaths in all industries or occupations. The asthma-COPD overlap PMRs were internally adjusted by 5-year age groups, sex, and race. CIs were calculated assuming Poisson distribution of the data. A PMR >1.0 indicates that there were more deaths associated with the condition in a specified occupation or industry than expected; a PMR <1.0 indicates that there were fewer deaths associated with the condition in a specified occupation or industry than expected.

^¶^ U.S. Census 2000 Occupation Classification System two-digit occupations with five or more deaths.

** Statistically significantly elevated PMR.

^††^ U.S. Census 2000 Occupation Classification System three-digit occupation groups with five or more deaths.

Among persons aged ≥65 years, industry and occupation data were available for 1,908 (98.3%) of 1,941 decedents with asthma-COPD overlap (624 [99.5%] of 627 men and 1,284 [97.7%] of 1,314 women). Asthma-COPD overlap PMRs were significantly elevated among men in certain industries (e.g., computer and electronic products [2.58]; lumber, wood products, and furniture [2.53]; agriculture, forestry, fishing and hunting [1.82]; beverage manufacturing [3.15]; and miscellaneous manufacturing [1.39)] and among women in private households (1.69), furniture and home furnishings stores (2.99), and unspecified food industries (3.72) (Table 1). By occupation, asthma-COPD overlap PMRs were significantly elevated among men in fishing, hunting, and forestry (3.78); farmers and farm managers (1.62); laborers and material movers (1.54); carpenters (1.68); and industrial production managers (2.23) and among women production workers (1.66) and waitresses (1.70) (Table 2).

## Discussion

Among persons aged ≥25 years, more women than men died from asthma-COPD overlap. A study using 2012 Behavioral Risk Factor Surveillance System data from South Carolina found that asthma-COPD overlap was more prevalent among women than among men (*2*). The annual age-adjusted death rate per million for both men and women decreased from 1999 through 2016. When analyzed separately, the age-adjusted death rate for asthma similarly declined among men and women from 1999 to 2016 (*3*). The age-adjusted death rate for COPD among men declined from 1999 to 2011; however, among women, it increased from 2000 to 2011 (*4*). A 2016 Danish study of the long-term prognosis of persons with chronic airway disease found that the number of deaths from chronic respiratory disease were higher among persons with asthma-COPD overlap with late-onset asthma than among those with COPD only (*5*).

The American Thoracic Society estimates that approximately 16% of asthma and 14% of COPD among adults is attributable to workplace exposures (*6*). Several workplace exposures, (e.g., dusts, secondhand smoke, welding fumes, and isocyanates) are causative agents for both asthma and COPD (*7*). An analysis of workplace exposures among U.S. adults using 2010 National Health Interview Survey data found that workers in industries and occupations similar to those identified in the current study had exposure to vapors, gas, dust, or fumes at work (*8*). In that study, an estimated 52.9% of workers in agriculture, forestry, fishing, and hunting and 42.8% of workers in manufacturing industries, as well as 61.5% of production workers, 50.8% of farming, fishing, and forestry workers, and 16.5% of adults in food preparation and serving occupations had frequent exposure to vapors, gas, dust, or fumes at work (*8*). Although cigarette smoking is the primary cause of COPD, 25% of U.S. adults with COPD have never smoked.*** Among nonsmoking adults in food preparation and serving occupations, an estimated 15.4% had frequent exposure to secondhand smoke at work (*8*). Exposure to these agents might explain the increased prevalence of asthma-COPD overlap mortality among workers in certain industries and occupations and should be considered for targets for public health interventions.

Nonpaid workers, nonworkers, and persons working at home had significantly elevated asthma-COPD overlap PMRs among both men and women aged 25–64 years, suggesting that asthma-COPD overlap might be associated with substantial morbidity resulting in loss of employment. Previous reports have similarly found that patients with asthma-COPD overlap were observed to have worse health outcomes than those with asthma or COPD alone (*1*,*5*). Moreover, persons with asthma caused or made worse by workplace exposures were similarly more likely to be unemployed and retire at a significantly younger mean age than were those with asthma that is not work-related (*9*). Retired and unemployed persons might have left the workforce because of severe asthma or COPD; however, complete decedent work histories were unavailable to assess such changes in employment.

The findings in this report are subject to at least five limitations. First, a discrete diagnosis code for asthma-COPD overlap does not currently exist, and no information was available to validate asthma and COPD diagnoses, which might be subject to misdiagnosis. A 1991 study from the United States found that 37% of subjects with a history of physician-diagnosed airways obstructive disease had airways obstructive disease reported on their death certificate, suggesting the potential for underreporting (*10*). In addition, it is possible that differences in patterns of asthma and COPD diagnosis regionally and over time might have affected how these diagnoses were recorded on death certificates. Second, discrete diagnosis codes for occupational asthma or COPD do not currently exist; therefore, determining whether the asthma or COPD diagnoses listed as underlying or contributing to death were caused by workplace exposures is not possible. Third, guidelines for reporting industry and occupation on death certificates^†††^ instruct recorders to report decedent’s “kind of business/industry” and “usual occupation” (i.e., “the type of job the individual was engaged in for most of his or her working life”). Therefore, if asthma and COPD were caused by workplace exposures, the industry and occupation reported on death certificates might not reflect those in which potential workplace exposures occurred. Workers might have changed jobs or held more than one job; however, information is not available to assess changes in employment. Fourth, no information was available to evaluate the smoking status of decedents, which might have caused or worsened the consequences of asthma or COPD. Finally, only selected states provided information on industry and occupation, and only for certain years; therefore, information by industry and occupation might not be nationally representative.

Among persons aged ≥25 years, deaths associated with asthma-COPD overlap were more frequent among women than among men. The association between asthma-COPD overlap mortality and nonworking status among adults of working age (25–64 years) suggests that asthma-COPD overlap might be associated with substantial morbidity resulting in loss of employment. Increased risk for asthma-COPD overlap mortality among adults in certain industries and occupations suggests targets for public health interventions (e.g., elimination or substitution of exposures, removing workers from exposures, engineering controls such as ventilation or enclosure of exposure generating processes, and workplace smoke-free policies) to prevent asthma and COPD in and out of the workplace. Continued surveillance for asthma-COPD overlap morbidity and mortality is essential to inform policy and intervention activities.

**Summary**

**What is already known about this topic?**

Patients with features of both asthma and chronic obstructive pulmonary disease (COPD), termed asthma-COPD overlap, have been reported to have worse health outcomes than those with asthma or COPD alone.

**What is added by this report?**

During 1999–2016, 18,766 U.S. decedents aged ≥25 years had asthma and COPD assigned on their death certificates as the underlying or contributing cause of death. Among adults aged 25–64 years, asthma-COPD overlap mortality was associated with nonworking status among men and women and bartending among women.

**What are the implications for public health practice?**

Excess risk for asthma-COPD overlap mortality among adults in certain industries and occupations suggests targets for public health interventions to prevent asthma and COPD in and out of the workplace.

## Footnotes

* https://www.cdc.gov/nchs/data_access/Vitalstatsonline.htm.

† Guidelines for reporting industry and occupation on death certificates instruct recorders to report decedent’s “kind of business/industry” and “usual occupation” (i.e., “the type of job the individual was engaged in for most of his or her working life”).

§ Colorado, Florida, Georgia, Hawaii, Idaho, Indiana, Kansas, Kentucky, Louisiana, Michigan, Nebraska, Nevada, New Hampshire, New Jersey, New Mexico, North Carolina, North Dakota, Ohio, Rhode Island, South Carolina, Texas, Utah, Vermont, Washington, West Virginia, and Wisconsin. States represent the state where the death took place, not necessarily where the decedent had resided.

¶ Age-adjusted death rates were calculated by applying age-specific death rates to the 2000 U.S. Census standard population age distribution. https://wonder.cdc.gov/wonder/help/mcd.html#Age-AdjustedRates.

** PMR was defined as the observed number of deaths from asthma-COPD overlap in a specified industry or occupation, divided by the expected number of deaths from asthma-COPD overlap. The expected number of deaths was the total number of deaths in industry or occupation of interest multiplied by a proportion defined as the number of asthma-COPD overlap deaths in all industries or occupations, divided by the total number of deaths in all industries or occupations. The asthma-COPD overlap PMRs were internally adjusted by 5-year age groups, sex, and race. CIs were calculated assuming Poisson distribution of the data. A PMR >1.0 indicates that there were more deaths associated with the condition in a specified occupation or industry than expected; a PMR <1.0 indicates that there were fewer deaths associated with the condition in a specified occupation or industry than expected.

†† *International Classification of Diseases, Tenth Revision* codes for asthma: J45.0 (predominantly allergic asthma), J45.1 (nonallergic asthma), J45.8 (mixed asthma), J45.9 (asthma, unspecified), J46 (status asthmaticus); and COPD: J40 (bronchitis, not specified as acute or chronic), J41.0 (simple chronic bronchitis), J41.1 (mucopurulent chronic bronchitis), J41.8 (mixed simple and mucopurulent chronic bronchitis), J42 (unspecified chronic bronchitis), J43.0 (MacLeod's syndrome), J43.1 (panlobular emphysema), J43.2 (centrilobular emphysema), J43.8 (other emphysema), J43.9 (emphysema, unspecified), J44.0 (chronic obstructive pulmonary disease with acute lower respiratory infection), J44.1 (chronic obstructive pulmonary disease with acute exacerbation, unspecified), J44.8 (other specified chronic obstructive pulmonary disease), J44.9 (chronic obstructive pulmonary disease, unspecified).

§§ https://surveillance.cancer.gov/joinpoint/.

¶¶ https://www.cdc.gov/niosh/topics/noms/default.html.

*** https://www.cdc.gov/tobacco/campaign/tips/diseases/copd.html.

††† https://www.cdc.gov/nchs/data/misc/hb_occup.pdf.
